# Sacred waste: queer ecology, urban abjection, and moral impurity in *Super Deluxe*

**DOI:** 10.3389/fsoc.2026.1888562

**Published:** 2026-08-24

**Authors:** Harsha Vasudevan, Akaitab Mukherjee

**Affiliations:** Vellore Institute of Technology - Chennai Campus, Chennai, India

**Keywords:** abjection (Kristeva), ecologies of impurity, queer ecology, super deluxe, urban ecocriticism

## Abstract

**Introduction:**

This study examines the film *Super Deluxe* through queer ecology to explore how contemporary Tamil cinema constructs queer subjectivity through the interplay of urban geographies, social stigma, and institutional moral regulation. Moving beyond traditional paradigm parameters of narrative visibility, this article investigates the physical environmental conditions that structure transgender experience in the film.

**Methodology:**

The study employs a qualitative methodology combining narrative and cinematic analysis to examine key scenes, dialogues, visual motifs, and spatial arrangements in *Super Deluxe*. Drawing on queer ecology, urban ecocriticism, and Julia Kristeva’s theory of abjection, the analysis investigates how cinematic space, bodily imagery, and urban environments shape the film’s representation of queerness.

**Results:**

The analysis shows that the film repeatedly situates queer existence within spaces marked by waste, bodily discomfort, secrecy, and social unease, particularly through the character of Shilpa, whose return as a transgender woman unsettles heteronormative assumptions surrounding family, gender, and respectability. At the same time, the film does not simply stigmatize queerness; rather, it redirects social e toward patriarchal masculinity, religious hypocrisy, and the normative structures that govern social legitimacy.

**Discussion:**

To explain this dynamic, the article introduces the concept of ecologies of impurity, which describes how bodies, spaces, affects, and moral discourses become entangled in the film’s critique of heteronormative order. By reading *Super Deluxe* through queer ecology, urban ecocriticism, and abjection, the study contributes to scholarship on Tamil queer cinema, environmental humanities, and South Asian film studies by showing how urban environments actively participate in the production and contestation of queer subjectivity.

## 1 Introduction

Representations of queer and transgender lives in Indian cinema have historically been shaped by heteronormative conventions that cast such identities as comic, deviant, pitiable, or socially disruptive rather than as fully realized subjects. Although the decriminalization of Section 377 in 2018 marked an important shift in public discourse around sexuality and queer visibility in India, cinematic transformation has been uneven. In recent years, however, regional cinemas, including Tamil cinema, have increasingly offered more layered portrayals of queer lives by foregrounding emotional vulnerability, familial conflict, stigma, and social exclusion rather than caricature alone. Films such as Peranbu (2018), Super Deluxe (2019), and Ka Pae Ranasingam (2020) indicate a notable shift in representational politics by situating gender and sexual difference within broader questions of care, kinship, marginality, and social morality. At the same time, scholarship on queer representation in Indian and Tamil cinema has largely concentrated on questions of identity, visibility, stigma, and narrative inclusion. Recent scholarship similarly observes that contemporary Indian cinema has increasingly moved beyond stereotypical portrayals to foreground questions of queer identity, representation, and social visibility (Meher, 2024). Labade and Ajgaonkar (2022) further argue that Indian regional cinemas have played a significant role in localising queer discourse by negotiating sexuality, identity, and cultural specificity beyond mainstream cinematic frameworks. Similarly, Nair and Francis (2024) demonstrate that audience responses to homosexuality in South Indian cinema remain deeply shaped by cultural norms, social attitudes, and heteronormative expectations, highlighting the continuing tensions surrounding queer visibility and acceptance. More recently, Thankalayam et al. (2026) argue that although queer representation in Indian cinema has become increasingly visible, significant challenges remain in moving beyond symbolic inclusion toward more nuanced and socially transformative representations. Recent work has further extended this discussion by examining how contemporary South Indian films negotiate family, heteronormativity, and cultural conservatism through nuanced portrayals of queer lives (Preethika Shree and Chithra, 2025). Collectively, these studies have been crucial in challenging exclusionary cinematic histories and advancing more nuanced understandings of queer lives in Indian and South Indian cinema. Yet this emphasis on representation has also meant that comparatively less attention has been paid to the material and spatial environments through which queer subjectivity is cinematically produced. In other words, while scholars have asked how queer characters are represented, whether they are granted dignity or agency, and how they disrupt dominant gender norms, there remains limited analysis of how films use urban space, domestic interiors, bodily discomfort, and institutional settings to shape queer life as affectively and morally charged.

Thiagarajan Kumararaja’s Super Deluxe (2019) offers a particularly compelling site for such an inquiry. Structured as a hyperlink film, *Super Deluxe* interweaves multiple narrative strands involving sexuality, secrecy, infidelity, religion, pornography, violence, and family within the urban milieu of Chennai. At the centre of the film is Shilpa, a transgender woman who returns to her family after having left home as a husband and father. Her return unsettles not only the domestic sphere but also the symbolic structures of masculinity, kinship, respectability, and normalcy through which the family has organized itself. More importantly, the film does not treat queerness simply as a matter of identity or visibility. Instead, it repeatedly places queer embodiment in relation to unstable urban and domestic environments, thereby linking sexuality to the material and moral disorder of everyday life.

While the critical corpus on *Super Deluxe* successfully unpacks its thematic investments in transgender visibility and formal hyperlink multiplicity, these text centric readings frequently bracket off the material realities of the film’s diegetic world. Rather than functioning merely as a narrative backdrop, the city’s streets, domestic interiors, institutional spaces, and public environments shape how queer subjectivity is experienced, regulated, and contested. This study targets this localized intersection, exploring how cinematic space, urban ecologies, and systemic moral panics map out the borders of queer vulnerability. To develop this argument, the article brings together queer ecology, urban ecocriticism, and Julia Kristeva’s theory of abjection. Queer ecology is useful because it challenges the naturalized binaries such as purity/impurity, normal/deviant, and natural/unnatural through which sexuality is often regulated. Urban ecocriticism extends this inquiry by reading the city as a lived ecology marked by waste, social exclusion, infrastructural instability, and unequal distributions of vulnerability. Abjection theory, in turn, helps explain how disgust, bodily excess, shame, and social expulsion operate in relation to queer embodiment. Read together, these frameworks make it possible to examine not only how Shilpa is represented, but how the film’s spaces, textures, and moral geographies shape the conditions through which queerness is made visible, stigmatized, and contested.

Within this framework, this study uses the term *Ecologies of Impurity* to describe the interconnected spatial, bodily, moral, and affective processes through which *Super Deluxe* associates queerness with moral disorder, and social excess while simultaneously destabilizing that association. The concept is not intended as a wholly separate theory, but as a synthetic analytical framework developed from queer ecology, urban ecocriticism, abjection theory, and cinematic spatial analysis. Rather than locating stigma in queer embodiment alone, “ecologies of impurity” names the broader network through which social exclusion circulates across bodies, domestic spaces, public institutions, religious spectacle, patriarchal masculinity, and the fragmented urban landscape of Chennai. It thus foregrounds how polluted urban spaces, humiliating institutional encounters, family shame, bodily discomfort, and moral panic become intertwined, such that contamination appears less as a property of queer embodiment than as an effect of heteronormative attempts to secure purity, order, and social legitimacy. This article argues that the film demonstrates how stigma circulates across bodies, spaces, and institutions rather than residing within queer embodiment alone. By bringing queer ecology, urban ecocriticism, and abjection theory into dialogue, the study shifts attention from representation to the spatial and ecological production of queer subjectivity and proposes Ecologies of Impurity as an integrative analytical framework for analysing contemporary Tamil urban cinema.

## 2 Literature review and conceptual framework

### 2.1 Queer representation, Tamil queer cinema, and existing readings of *Super Deluxe*

Historically, South Asian film scholarship has traced a rigid representational politics wherein queer bodies are relegated to marginality or comic relief. However, contemporary regional media landscapes particularly within Tamil cinema have begun dismantling these tropes by framing gender and sexual nonconformity within complex domestic webs of kinship, precarity, and emotional negotiation rather than relying on flat caricatures. Within Tamil cinema specifically, transgender representation has undergone a visible transformation over the past two decades. Earlier portrayals often reduced transgender figures to spectacles of humour, fear, or pity, reproducing stigma rather than challenging it. More recent films, however, increasingly seek to humanize queer and transgender experience by attending to questions of belonging, emotional survival, kinship, and social acceptance. It is within this broader shift that Super Deluxe (2019) has received critical attention for its portrayal of Shilpa, a transgender woman whose return to her family unsettles conventional assumptions surrounding masculinity, parenthood, morality, and social respectability. Existing discussions of the film emphasize its refusal to frame transgender identity solely through victimhood or sensationalism while also examining its fragmented narrative structure, symbolic characterization, and critique of dominant social norms (Murali, 2024; Mishra, 2021; Sundar, 2021). Mishra (2021), for instance, reads the film in relation to emotional acceptance and the instability of rigid gender binaries, while Sundar (2021), by contrast, focuses on the film’s hyperlink structure, narrative fragmentation, and anti-linear storytelling, arguing that such formal strategies destabilize moral certainty and expose the contradictions underlying normative social systems. Similarly, Nandakumar and Jenitha (2023) examine how the film interrogates dominant moral values through its interconnected narratives, highlighting its critique of social conformity and ethical judgment. Likewise, Shageel (2020) interprets *Super Deluxe* as a critique of entrenched social norms through its interconnected narratives and unconventional characterization, demonstrating how the film challenges normative assumptions surrounding gender, sexuality, and morality. These baseline studies provide vital analytical platforms, yet they leave an open question regarding the material and environmental mechanics of this subversion. For instance, Mishra's (2021) examination of queer kinship can be structurally extended by asking how these domestic bonds are physically compressed or sustained within the film’s claustrophobic interior sets. Similarly, Sundar’s (2021) critique of the film’s anti-linear narrative structures can be advanced by parsing how this formal fragmentation mirrors the material degradation and infrastructural decay of the postcolonial city itself. By shifting focus from what Shilpa symbolically represents to how her movements are spatialized, this paper systematically builds upon these existing foundations by examining the material, spatial, and ecological production of queer subjectivity.

### 2.2 Queer ecology, urban ecocriticism, abjection, and cinematic space

To address this gap, the present study brings together queer ecology, urban ecocriticism, abjection theory, and spatial approaches to cinema in order to examine how *Super Deluxe* links queer embodiment to waste, and moral disorder within the urban environment. Although these frameworks emerge from different disciplinary traditions, they converge around a central concern: the relationship between bodies, environments, social regulation, and the politics of exclusion. Queer ecology provides an important starting point because it interrogates the ideological association between heterosexuality and “naturalness,” while exposing how queer desires and nonnormative bodies are often constructed as excessive, impure, or ecologically disruptive. Mortimer-Sandilands and Erickson (2010) argue that ecological discourse has frequently naturalized heterosexuality by positioning reproductive, orderly, and socially sanctioned bodies as closer to nature, while marking queer life as unnatural or contaminating. Similarly, Gaard (1997) demonstrates that ecological oppression and sexual oppression are often intertwined through patriarchal systems that regulate bodies, reproduction, purity, and legitimacy. Queer ecology is therefore useful for understanding how ideas of order, cleanliness, naturalness, and social legitimacy become attached to some forms of embodiment while others are cast as excessive or contaminating. In the context of *Super Deluxe*, this framework illuminates how Shilpa’s return unsettles the normative family and how transgender existence is positioned within the film’s social world as a disturbance to patriarchal and familial order. However, queer ecology alone does not fully account for the film’s repeated emphasis on polluted urban spaces, discarded environments, bodily fluids, death, pornography, and infrastructural decay. Urban ecocriticism extends the analysis by shifting ecological attention from wilderness or nature to the city as a lived environment shaped by waste, overcrowding, infrastructural instability, and unequal distributions of vulnerability. Buell (2001) and Garrard (2012) both emphasize that urban spaces are not environmentally neutral but are marked by symbolic and material geographies of exclusion, where dirt, pollution, waste, and decay often become attached to marginalized communities and unstable identities. Urban ecocriticism is therefore especially relevant to *Super Deluxe*, where Chennai repeatedly appears through claustrophobic interiors, fragmented streets, dimly lit motel rooms, pornography networks, bodily fluids, and scenes of violence and death. Such spaces do not function merely as visual backdrops; they become active environments through which emotional repression, patriarchal violence, secrecy, and social hypocrisy are spatially organized. Kristeva’s theory of abjection provides a third key lens for understanding how abjection functions in the film. In *Powers of Horror* (1982), Kristeva describes abjection as the process through which social order is maintained by rejecting what appears impure, excessive, or threatening to stable boundaries of self and society. Bodily fluids, corpses, decay, waste, and ambiguous identities become abject because they disturb distinctions between self and other, order and disorder. Abjection theory has been especially influential in queer studies because heteronormative societies have often associated queer existence with disgust, bodily excess, secrecy, symbolic pollution, and moral panic. Butler’s (1993) work similarly shows how normative identities sustain themselves by excluding and stigmatizing bodies deemed unintelligible or disruptive. In *Super Deluxe*, abjection is not confined to the symbolic status of Shilpa’s body; it circulates through recurring images of death, pornography, blood, bodily discomfort, humiliation, and urban decay. Shilpa’s presence destabilizes normative expectations of masculinity, fatherhood, domesticity, and familial purity, yet the film also reveals that patriarchal violence, domestic repression, and religious hypocrisy are themselves deeply contaminating.

A spatial approach to cinema further strengthens this framework by foregrounding the role of environments, architecture, framing, movement, and social location in the production of meaning. Lefebvre (1991) argues that space is socially produced through systems of surveillance, regulation, exclusion, and power rather than existing as a neutral backdrop. In film studies, this means that domestic interiors, public streets, institutional corridors, motel rooms, and urban nightscapes must be understood not simply as settings but as ideological and affective structures that shape how characters are seen, controlled, and made vulnerable. In queer cinema, space often functions as a site of concealment, surveillance, negotiation, and resistance. Gopinath (2005) has shown that queer South Asian cultural narratives frequently use fragmented spatial environments to register the instability of queer existence within heteronormative social structures. This is especially relevant to *Super Deluxe*, whose hyperlink narrative intensifies spatial fragmentation by connecting multiple lives across overlapping urban sites of secrecy, regulatory violence, and emotional collapse. The convergence of these distinct theoretical lenses offers a multi-scalar framework for reading the text. While queer ecology problematizes the naturalized constructs of heteronormative space, urban ecocriticism anchors this critique in material urban infrastructures, and abjection theory tracks the psychological and visceral fallout of these spatial crossings. Together, they form an integrated toolkit for exploring how architectural containment and material atmospheres shape marginalized subjectivities.

### 2.3 Research gap and conceptual intervention: ecologies of impurity

To synthesize these overlapping intersections, this study introduces the concept of *ecologies of impurity*. Rather than positing a new standalone ontology, this framework acts as an integrative critical heuristic. It maps out the systemic circuits through which somatic discomfort, structural waste, institutional exclusion, and moral surveillance flow through a shared urban network. While traditional spatial analyses often focus on geographic boundaries, an ecological process tracks the relational traffic of stigma. In *Super Deluxe*, contamination is not an essentialized trait located within Shilpa’s trans body; instead, it is a dynamic currency that circulates across domestic households, police stations, religious altars, and urban ruins. By deploying this concept, the article highlights how heteronormative structures attempt to maintain an illusion of social hygiene by casting nonconforming subjects as pollutants, while simultaneously demonstrating how those very regulatory institutions are internally fractured by their own moral decay. Accordingly, this study makes two principal contributions. First, it extends scholarship on Tamil queer cinema beyond representational analysis by demonstrating how cinematic environments and urban materiality actively shape queer subjectivity. Second, it contributes to queer ecology and urban film studies by conceptualizing exclusion as a spatial and affective process linking waste, shame, institutional regulation, and social exclusion.

## 3 Methodology

### 3.1 Research design and scene selection

This study adopts an interpretive qualitative research design that combines narrative analysis, visual semiotics, and cinematic analysis to examine urban space, queer embodiment, and moral discourse in Thiagarajan Kumararaja’s Super Deluxe (2019). The film is treated as a cultural and cinematic text in which meaning is produced through the interaction of narrative structure, character performance, visual composition, spatial arrangement, dialogue, and symbolic imagery. The study therefore focuses not only on what the film represents, but also on how cinematic form and urban environment participate in the construction of queer precarity, abjection, and social exclusion. To ensure methodological transparency, the primary unit of analysis is defined as the scene, understood here as a discrete cinematic segment marked by continuity of setting, narrative action, and character interaction, and bounded by a shift in location, time, or focal interaction. Although the film was viewed in its entirety, the detailed analytical corpus was delimited through a purposive scene-selection strategy. This strategy was used to identify scenes most relevant to the study’s central concerns: queer embodiment, abjection, institutional exclusion, and moral regulation.

The selected scenes were evaluated using the following inclusion criteria:

- scenes featuring Shilpa that explicitly portray transgender visibility, spatial negotiation, or domestic and public encounters;
- scenes foregrounding Chennai’s urban environment, including domestic interiors, transit spaces, motel rooms, or spaces associated with waste, decay, or infrastructural instability;
- scenes depicting institutional surveillance, religious performance, patriarchal policing, bodily discomfort, humiliation, or moral judgement; and
- scenes in which cinematic techniques such as framing, lighting, colour, or sound intensify themes of exclusion, shame, precarity, or social regulation.

The exclusion criteria were as follows:

- non-diegetic transitions or brief montage sequences that did not include significant character interaction or thematic development;
- hyperlink segments that functioned only as narrative bridges without contributing substantially to the study’s focus on space, queerness, morality, or social exclusion; and
- scenes that repeated previously coded motifs without adding new analytical insight.

### 3.2 Data collection and analytical procedure

To strengthen analytical consistency and procedural transparency, the film was subjected to six sequential viewing stages, each corresponding to a different phase of data collection and interpretation.

*First viewing (familiarization)*: An initial full viewing was undertaken to understand the film’s overall hyperlink structure, narrative trajectories, and central character arcs. This stage helped identify the major thematic clusters relevant to queer embodiment, urban disorder, family conflict, religious morality, and social shame.

*Second viewing (structural mapping)*: During the second viewing, the film was segmented into scene-based analytical units. Timestamps were logged and formal boundaries were established for the 45 selected scenes based on shifts in setting, interaction, or narrative emphasis.

*Third viewing (dialogue and transcription)*: The selected scenes were revisited to record key diegetic Tamil dialogues alongside their corresponding English subtitles. This stage was important for preserving linguistic nuance and for identifying recurring verbal patterns related to shame, normalcy, morality, disgust, respectability, and social regulation. Where relevant, attention was also paid to tone, address, and scene context in order to preserve interpretive nuance in translation.

*Fourth viewing (visual and semiotic notation)*: The fourth viewing focused on non-verbal cinematic elements, including framing, camera distance, lighting, colour palette, mise-en-scène, character body language, gesture, silence, and ambient sound. Particular attention was paid to how these elements shaped the emotional and spatial atmosphere of each scene.

*Fifth viewing (coding and thematic indexing)*: At this stage, the observation notes, transcripts, and scene descriptions were entered into a *scene-based coding matrix*. For each scene, the matrix recorded the timestamp, narrative context, principal characters, setting, relevant dialogue, visual motifs, and preliminary interpretive codes.

*Sixth viewing (comparative review and refinement)*: A final viewing was undertaken to compare the coded scenes across the corpus, refine code definitions, and verify interpretive consistency. This stage helped ensure that thematic categories were not assigned on the basis of isolated moments but were supported by recurring visual, dialogic, and spatial evidence across the film.

### 3.3 Hybrid coding and theme development

The scene-based analysis and thematic interpretation were conducted by the first author using a hybrid thematic approach that combined deductive and inductive analysis. Deductively, the analysis was informed by queer ecology, urban ecocriticism, abjection theory, and spatial approaches to cinema. These frameworks provided sensitizing concepts rather than fixed interpretive categories, allowing the coding process to begin with theoretically relevant concerns such as stigma, disgust, waste, spatial exclusion, bodily precarity, and heteronormative regulation. At the same time, the coding process remained inductive and scene-responsive. During repeated viewing, patterns specific to the film emerged from its own narrative and visual texture, particularly in relation to family shame, institutional hostility, masculine insecurity, religious spectacle, and urban precarity. These recurrent motifs were incorporated into the coding process only when they were consistently supported by multiple scenes and by more than one type of cinematic evidence, such as dialogue, setting, body language, or visual composition. The initial coding stage included scene-level descriptive and interpretive labels such as urban waste and decay, queer abjection, institutional exclusion, religious spectacle, masculine anxiety, social respectability, and moral shame. However, these labels were not treated as fixed categories from the outset. Instead, they were revised through iterative comparison across scenes. Some codes were merged, narrowed, or redefined depending on how they functioned within the film’s narrative and spatial logic. For example, scenes initially coded under urban waste were re-examined to distinguish between physical environmental disorder, symbolic, and the affective atmosphere of urban precarity. Likewise, scenes coded as queer abjection were compared to determine whether the abject dynamic was produced through public disgust, familial rejection, bodily discomfort, institutional humiliation, or moral condemnation.

### 3.4 Operationalizing the interdisciplinary framework

To make the interdisciplinary framework analytically workable, the study uses an integrated analytical matrix linking four complementary theoretical and analytical lenses to specific cinematic and thematic indicators in the film. Queer ecology is used to analyse how the film constructs and destabilizes normative ideas of naturalness, purity, domestic order, and reproductive legitimacy. In cinematic terms, this includes scenes in which family structures, domestic roles, and heteronormative expectations are unsettled by Shilpa’s return and by the film’s treatment of intimacy, shame, and social normalcy.

*Abjection theory* is used to examine disgust, bodily excess, boundary anxiety, and social expulsion. This lens is applied to scenes involving bodily discomfort, blood, death, pornography, humiliation, or moments in which queer embodiment is framed as disruptive, excessive, or socially contaminating.

*Urban ecocriticism* is used to analyse the material and symbolic dimensions of Chennai’s urban environment, including infrastructural decay, cluttered interiors, waste circulation, motel spaces, compromised domestic settings, and other sites of abandonment or disorder. This framework helps connect environmental instability to social precarity and moral regulation.

*Film and semiotic analysis* are used to interpret the formal cinematic construction of atmosphere and meaning through framing, colour, lighting, camera distance, sound, silence, and mise-en-scène. Drawing on principles of visual grammar (Kress and van Leeuwen, 2021) alongside established approaches to film analysis (Bordwell and Thompson, 2019), these elements are treated not as aesthetic background, but as active components in the film’s production of tension, shame, secrecy, and exclusion. Global South queer studies contextualise the film within contemporary postcolonial India by examining how family structures, religious morality, institutional authority, and public norms shape the regulation of queer embodiment. This lens enables the analysis to interpret cinematic representations within their specific socio-cultural context, emphasizing how queer identities are negotiated through culturally specific histories of nation, kinship, respectability, and postcolonial social norms (Rao, 2020).

### 3.5 Analytical credibility and reflexive validation

The scene selection, repeated viewing, scene-based analysis, and thematic interpretation were conducted by the first author. To strengthen interpretive credibility, transparency, and analytical consistency, the study employed several qualitative strategies throughout the research process. A process of reflexive validation was maintained throughout the six stages of viewing. An analytical journal was used to record emerging interpretations, theoretical assumptions, and shifts in analytical decisions so that the influence of prior conceptual expectations could be made visible and critically monitored rather than left implicit. Constant scene comparison made it possible to examine whether analytical categories such as masculine anxiety, social shame, or institutional exclusion functioned consistently across different narrative strands and settings rather than being inferred from isolated scenes alone. A digital audit trail was maintained linking each analytical category to its corresponding timestamp, dialogue excerpt, scene description, visual note, and analytical rationale. This documentation strengthens the transparency of the analytical process by demonstrating how thematic interpretations were systematically derived from specific cinematic evidence. Finally, the study uses theoretical triangulation by reading the same scene corpus through queer ecology, urban ecocriticism, abjection theory, cinematic analysis, and Global South queer studies. Rather than assuming that theory automatically determines meaning, this comparative approach was used to test whether the interpretation of a scene was supported simultaneously by narrative, visual, spatial, and cultural evidence. To reduce the risk of confirmation bias, theory-informed analytical categories were not treated as fixed explanatory truths; instead, scenes were revisited comparatively, and categories were retained only when they were supported by recurring patterns across multiple scenes and across multiple forms of cinematic evidence. No theme was retained solely on the basis of theoretical relevance; rather, themes were retained only when they recurred across multiple scenes and were supported by more than one form of cinematic evidence, including dialogue, spatial setting, visual composition, or embodied performance. In this way, the analysis remains grounded in the film’s textual and visual material while still enabling the broader conceptual intervention proposed in the article.

## 4 Findings

The scene-based qualitative analysis of *Super Deluxe* identifies a recurring set of patterns through which queerness, urban disorder, bodily discomfort, and moral anxiety become spatially and affectively entangled across the film’s hyperlink narrative. Rather than presenting marginalization as a stable attribute of queer identity, the film repeatedly stages it through environments, institutions, and social interactions that frame Shilpa’s return as disruptive to domestic order, public respectability, and heteronormative legitimacy.

**Figure 1:**
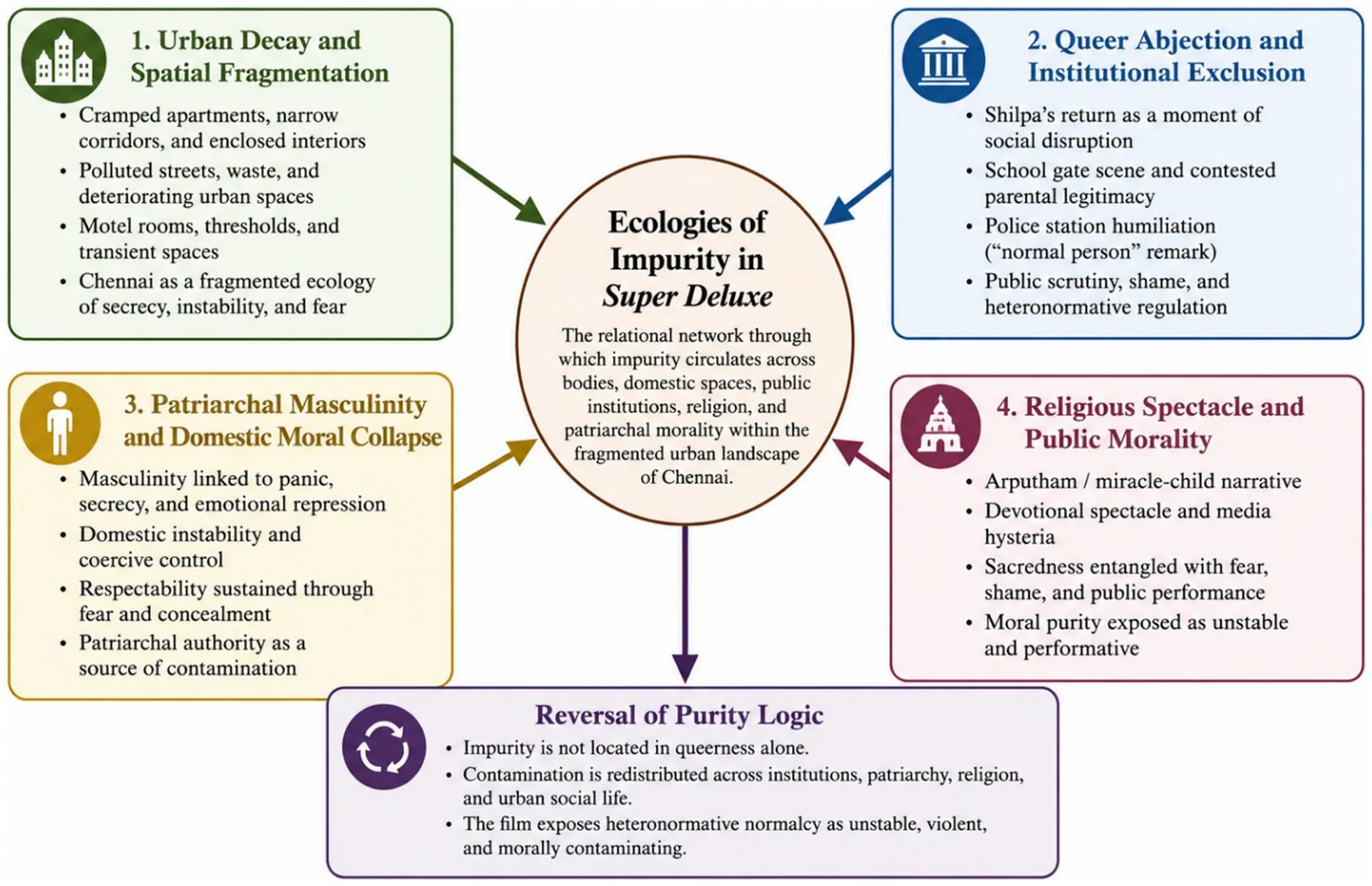
Conceptual thematic map of ecologies of impurity in *Super Deluxe*. The figure Illustrates the four major empirical clusters identified in the analysis and how they converge to produce an ecology of impurity. Through this ecology, the film progressively displaces exclusion from queer embodiment and redirects it toward the social structures that claim moral authority.

As summarized in Figure 1, these findings emerge through four interconnected empirical clusters that together demonstrate how exclusion is progressively displaced from queer embodiment toward the institutions and moral structures that regulate social life.

### 4.1 Urban decay and spatial fragmentation

One of the clearest empirical patterns concerns the film’s construction of Chennai as a fractured urban ecology marked by confinement, waste, infrastructural instability, and emotional suffocation. The selected scenes repeatedly situate key moments of conflict within cramped apartments, narrow corridors, deteriorating motel rooms, polluted streets, institutional thresholds, and cluttered interiors rather than within stable or restorative domestic spaces. Shilpa’s return sequence is especially important in this regard. Her arrival is not framed within a private moment of familial reunion, but through the tense social architecture of the apartment complex: neighbours gather, faces register shock, the staircase and corridor become crowded points of surveillance, and the domestic threshold itself is transformed into a public site of scrutiny. The scene’s fragmented framing and obstructed sightlines make the apartment corridor function less as a homecoming space than as an arena of social judgment. This spatial logic recurs elsewhere in the film through motel rooms, police stations, school gates, traffic-filled streets, and dim interiors that repeatedly stage emotional vulnerability within environments of instability rather than comfort. The cumulative effect is to present Chennai not merely as a backdrop to queer life but as a recurring environment in which secrecy, humiliation, and compromised belonging are repeatedly staged.

### 4.2 Queer abjection and institutional exclusion

The film’s treatment of Shilpa further intensifies this ecological process by linking queer embodiment to public discomfort, familial uncertainty, and institutional exclusion. Several scenes foreground the difficulty of securing legibility for Shilpa within heteronormative structures of family and social order. In the school sequence, for example, the watchman’s refusal to accept Shilpa as Raasukutti’s parent produces a moment in which kinship itself is subjected to bureaucratic suspicion. The scene is visually organized around the school gate, which separates the child from institutional recognition and turns the entrance into a symbolic boundary of legitimacy. The emotional force of the sequence lies not only in the dialogue but also in the framing of Raasukutti’s insistence against the watchman’s suspicion, which reveals how public institutions regulate belonging through rigid assumptions about gender and parenthood. A similar logic operates in the police station sequence, where the officer’s instruction to “take a normal person along” explicitly marks Shilpa as outside the category of social normalcy. The violence of the remark is intensified by the ordinariness of the setting: background movement continues, ambient police noise remains uninterrupted, and no dramatic visual break isolates the insult. Instead, discrimination is absorbed into the everyday functioning of institutional life. Through such scenes, the film associates queer embodiment with abjection not because Shilpa is inherently excessive or contaminating, but because institutions repeatedly position her as unintelligible, embarrassing, or socially improper.

### 4.3 Shared abjection and material circulation

At the same time, the film does not confine abjection to Shilpa’s body alone. It distributes impurity across a wider network of bodily discomfort, death, pornography, humiliation, secrecy, and emotional panic that cuts across the hyperlink narrative. Recurring motifs of blood, corpses, pornography, physical exhaustion, and polluted interiors create an atmosphere in which bodily excess becomes inseparable from moral instability. Yet the film’s use of these motifs does not simply reinforce the idea that queer life is socially impure. Instead, it places queer embodiment within a broader cinematic world already saturated by abject materiality. The repeated presence of waste-filled streets, decaying rooms, bodily fluids, and emotionally claustrophobic interiors establishes a broader field of abject materiality before impurity is explicitly projected onto Shilpa. This is especially significant because it prevents the narrative from isolating transgender identity as the singular site of contamination. Shilpa certainly becomes the target of disgust, suspicion, and humiliation, but the film repeatedly situates those reactions within a larger ecology of bodily and moral disorder that exceeds her and implicates the world around her.

### 4.4 Patriarchal masculinity and domestic moral collapse

A further recurring pattern emerges through the representation of patriarchal masculinity as unstable, performative, and itself contaminating. Across the film’s different narrative strands, male authority is repeatedly linked to secrecy, panic, violence, emotional repression, and moral collapse rather than to coherence or legitimacy. This becomes visible in scenes where domestic respectability is sustained through concealment, aggression, or fear, and where men struggle to maintain control over family, sexuality, or public appearance. For instance, the confrontation involving Arputham after the “miracle” child episode, together with the motel-room storyline involving Vaembu, illustrates how male authority is repeatedly expressed through panic, concealment, and emotional volatility rather than confidence or moral certainty. Rather than presenting patriarchal masculinity as the stable opposite of queer disruption, the film exposes it as one of the principal generators of disorder within the narrative. Visually, this is reinforced through tense domestic interiors, abrupt confrontations, emotionally volatile performances, and the repeated collapse of authoritative male self-control. Masculinity in the film is therefore not associated with moral clarity but with shame, denial, and coercive attempts to restore order. This is crucial to the film’s reversal of social regulation, because the source of social exclusion gradually shifts away from Shilpa’s gender nonconformity and toward the fragile systems of patriarchal control that seek to manage it.

### 4.5 Religious spectacle and public morality

The film extends this critique through its treatment of religion, public morality, and spectacle. The narrative involving Arputham and the “miracle” child repeatedly stages religion not as a space of ethical stability, but as a chaotic performance structured by fear, spectacle, and moral irrationality. Devotional gatherings, media circulation, public obsession, and emotionally exaggerated reactions transform spirituality into a form of spectacle rather than compassion. The visual construction of these scenes overcrowded interiors, artificial lighting, loud devotional soundscapes, and claustrophobic framing creates an atmosphere of confusion and hysteria rather than transcendence. Importantly, these scenes are juxtaposed with pornography, secrecy, violence, and emotional collapse elsewhere in the film, destabilizing any clean distinction between the sacred and the contaminated. Religion thus appears as another institution through which exclusion is displaced, denied, and theatrically managed. The same applies to public morality more broadly: the neighbourhood corridor, the school gate, and the police station all function as sites where normative social order attempts to classify bodies, regulate kinship, and enforce respectability, yet each of these spaces is itself marked by humiliation, fear, or hypocrisy.

### 4.6 The role of cinematic form

Cinematic form plays an important role in binding these patterns together. The film repeatedly uses fragmented framing, spatial obstruction, low-key lighting, neon colour contrasts, and restrained sound design to produce an atmosphere of unease rather than moral resolution. In scenes involving Shilpa and Raasukutti, soft low lighting and extended silences create emotional intimacy while also underscoring the instability of socially recognizable kinship. By contrast, institutional spaces such as the police station are often more brightly lit and acoustically busy, which intensifies the normalization of humiliation rather than alleviating it. The recurrence of dim corridors, shadow-filled rooms, neon-lit interiors, and abrupt shifts between domestic, institutional, and public spaces contributes to a sensory environment in which secrecy, exposure, panic, and judgment are constantly in circulation. Together, these cinematic techniques reinforce the recurring empirical patterns identified across the selected scenes and prepare the ground for the more detailed interpretive analysis presented in the following section.

### 4.7 Summary: reversal of the purity logic

Taken together, these empirical patterns support the article’s central claim that *Super Deluxe* constructs queer life through what this study terms ecologies of impurity. The film initially appears to align Shilpa with contamination by placing her within scenes of social discomfort, institutional rejection, and public scrutiny. However, when the scenes are read comparatively, a different pattern emerges: exclusion is shown to circulate most forcefully through the very structures that claim to protect normalcy patriarchal masculinity, domestic respectability, religious spectacle, institutional regulation, and the fragmented urban environments that sustain them. The significance of the film’s queer ecological politics lies precisely in this displacement. Rather than allowing queerness to remain the object of disgust, *Super Deluxe* gradually reveals that the more pervasive moral disorder resides in the violence, hypocrisy, surveillance, and moral panic through which heteronormative social order attempts to secure its own purity. As mapped visually in Figure 1, the film does not simply represent queer abjection; it redistributes impurity across bodies, spaces, and institutions, transforming contamination from a stigma attached to Shilpa into a critical lens for reading the instability of contemporary urban social life.

## 5 Analysis

The choice of narrative analysis in this study is particularly significant because *Super Deluxe* operates through fragmented storytelling, intersecting lives, and emotionally unstable urban environments that cannot be adequately examined through conventional textual interpretation alone. Unlike purely thematic approaches, narrative analysis enables a deeper exploration of how cinematic space, bodily representation, visual symbolism, and emotional tension collectively construct meanings surrounding queerness, masculinity, and social disorder. The method is especially effective for analysing the film’s hyperlink narrative structure, where violence, shame, desire, secrecy, and moral collapse circulate across interconnected storylines and urban spaces. Furthermore, narrative analysis aligns closely with the study’s queer ecological framework, as it allows the examination of how bodies, environments, institutions, and social relationships become entangled within broader structures of contamination, exclusion, and heteronormative control. Through this approach, the study critically investigates how *Super Deluxe* transforms urban fragmentation and moral instability into a cinematic critique of contemporary social order.

### 5.1 Enhanced interpretive depth through cinematic techniques

By integrating cinematography, lighting, body language, and sound design into the thematic analysis, this study examines how *Super Deluxe* constructs queer existence not only through dialogue and narrative but also through visual and sensory environments. The film repeatedly uses fragmented urban spaces, emotionally charged silences, oppressive interiors, and unstable visual compositions to communicate social discomfort surrounding gender nonconformity, sexuality, masculinity, and moral anxiety. Rather than functioning merely as aesthetic choices, these cinematic techniques become central to the film’s critique of heteronormative morality and urban social disorder.

#### 5.1.1 Cinematography and spatial framing

The film employs spatial confinement and fragmented framing to visually communicate emotional suffocation and social alienation. One of the earliest examples appears during Shilpa’s return sequence, where the camera repeatedly cuts to shocked faces, hesitant glances, and obstructed reaction shots from neighbours witnessing her arrival. The scene avoids stable framing and instead creates visual discomfort through abrupt cuts and fragmented compositions, reinforcing the social instability generated by gender nonconformity. The narrow staircase, crowded apartment corridor, and tense domestic interior collectively transform the neighbourhood space into an environment of surveillance and judgment. Similarly, the school sequence involving Raasukutti and Shilpa intensifies this spatial tension. When the watchman refuses to believe that Shilpa is Raasukutti’s father, the scene is framed within the school gate, visually separating the child from institutional acceptance. The gate itself becomes symbolic of exclusion, while the camera alternates between Raasukutti’s emotional insistence and the guard’s suspicious gaze. This framing reflects how social institutions regulate familial legitimacy through rigid gender expectations. The film’s hyperlink narrative structure further contributes to spatial fragmentation by constantly shifting between motel rooms, police stations, pornography networks, hospitals, traffic-filled streets, and cramped apartments. Taken together, these abrupt shifts between corridors, school spaces, police settings, motel rooms, and domestic interiors present Chennai as a fragmented urban environment in which experiences of shame, surveillance, secrecy, and emotional tension recur across multiple narrative strands.

#### 5.1.2 Lighting and symbolic contrast

Thiagarajan Kumararaja’s visual storytelling makes extensive use of a carefully controlled neon colour palette dominated by green, blue, and red tones. Rather than assigning fixed symbolic meanings to these colours, this analysis examines how they function within the film’s *mise-en-scène* to shape emotional atmosphere, spatial relationships, and narrative tension. Following approaches to cinematic semiotics, visual grammar, and mise-en-scène (Kress and van Leeuwen, 2021; Bordwell and Thompson, 2019), lighting and colour are interpreted contextually in relation to framing, narrative progression, and character interaction rather than through universal symbolic meanings. Several scenes involving Shilpa unfold within dimly lit interiors and shadow-filled spaces that visually reinforce her social marginalization. During the emotionally significant “Dad or Mom?” conversation between Raasukutti and Shilpa, soft low lighting creates emotional intimacy while simultaneously reflecting uncertainty surrounding familial identity and gender classification. In contrast, scenes associated with public masculinity and institutional authority often appear under brighter and harsher lighting. For instance, the police station sequence where the officer tells Shilpa to “take a normal person along” is presented within a cold, brightly illuminated setting, intensifying the normalized violence of social discrimination. Across multiple scenes, the film repeatedly employs saturated green, blue, and red lighting in conjunction with specific narrative situations, spatial arrangements, and character interactions. Following a cinematic semiotic approach, these recurring visual patterns are interpreted in relation to their contextual use within the film rather than as fixed symbolic meanings attached to individual colours. In scenes involving Shilpa and Raasukutti, softer blue and low-lit tones often accompany moments of hesitation, emotional vulnerability, or intimate conversation, such as the “Dad or Mom?” exchange. By contrast, the police-station sequence is staged in a brighter, harsher visual register that makes the officer’s “normal person” remark feel administratively routine rather than dramatically exceptional. Elsewhere, red and neon-lit interiors recur in scenes associated with panic, secrecy, sexual exposure, or emotional excess, especially in narrative strands linked to pornography, motel spaces, and confrontation. Rather than assigning a single stable meaning to each colour, the film uses lighting contrast to differentiate emotional registers across spaces and to intensify the atmosphere of unease, exposure, and instability that runs through the city. Taken together, these lighting contrasts encourage viewers to question the apparent neutrality of institutional authority, patriarchal masculinity, and normative social order, suggesting that the film locates moral contradiction not in queer embodiment but within the structures that regulate it. The recurring lighting contrasts discussed above demonstrate how colour, framing, and mise-en-scène work together to produce emotional atmosphere and reinforce the film’s representation of social tension and instability.

#### 5.1.3 Body language and emotional suppression

Body language functions as a major emotional language throughout the film, often communicating discomfort and repression more powerfully than dialogue itself. During Shilpa’s initial return, the surrounding characters avoid sustained eye contact, physically distance themselves, and display visible bodily hesitation, reflecting the social unease generated by her transformed identity. Shilpa herself frequently lowers her gaze, pauses before speaking, and moves cautiously through domestic spaces, revealing the psychological burden of social scrutiny. A particularly significant contrast emerges through Raasukutti’s physical interactions with Shilpa. Unlike the adults around him, the child approaches Shilpa without visible disgust or fear. His relaxed posture, emotional openness, and affectionate proximity destabilize the socially learned nature of prejudice. This becomes especially powerful during the final emotional confrontation where Raasukutti tells Shilpa:

*“Be a man or be a woman, just be with us.”*

The emotional force of the scene emerges not only through dialogue but through the child’s physical closeness, tears, and refusal to reject Shilpa’s identity. The scene challenges heteronormative assumptions surrounding parenthood and demonstrates how acceptance becomes emotionally embodied through gesture and intimacy. In contrast, several male characters throughout the film display rigid posture, aggressive reactions, emotional withdrawal, and impulsive violence. These bodily performances suggest the fragility of patriarchal masculinity, particularly in scenes where emotional repression gives way to panic, shame, or aggression.

#### 5.1.4 Sound design and emotional undercurrents

The film’s sound design reinforces emotional instability through silence, ambient urban noise, and abrupt tonal disruptions. Rather than relying excessively on melodramatic background music, many emotionally tense scenes unfold through restrained soundscapes dominated by traffic noise, television audio, distant conversations, sirens, and environmental disturbance. This creates a sense of psychological unease that mirrors the instability of the urban environment itself. One striking example occurs during the police station sequence involving Shilpa and Raasukutti. The surrounding ambient sounds of police activity, movement, and indifferent conversation continue uninterrupted even after the humiliating “normal person” remark is delivered. The absence of dramatic musical emphasis makes the discrimination appear normalized within everyday institutional life, thereby intensifying the emotional violence of the scene. Similarly, several emotionally vulnerable moments between Shilpa and Raasukutti are accompanied by subdued soundscapes and extended silences that foreground emotional intimacy over dramatic spectacle. The film repeatedly allows silence to communicate fear, shame, exhaustion, and longing, particularly in scenes where characters struggle to articulate emotions constrained by social expectations. Through these layered auditory techniques, *Super Deluxe* uses ambient sound and silence to reinforce emotional tension, institutional indifference, and the broader atmosphere of fragmentation that shapes its urban world.

#### 5.1.5 Religious spectacle and the politics of moral purity

The narrative involving Arputham and the miraculous child becomes central to *Super Deluxe’s* critique of religious morality, collective fear, and purity-based social systems. Rather than presenting religion as a source of ethical stability, the film portrays spirituality as deeply entangled with media sensationalism, emotional manipulation, and public spectacle. Scenes involving miracle narratives, devotional gatherings, televised spirituality, and public hysteria are repeatedly juxtaposed with violence, pornography, secrecy, and emotional collapse, destabilizing the distinction between sacredness. Religion within the film therefore operates less as a space of compassion and more as a performative structure sustained through spectacle, surveillance, and moral anxiety. This instability becomes particularly visible through the film’s chaotic visual construction of religious spaces. Artificial lighting, overcrowded interiors, loud devotional chanting, emotionally exaggerated reactions, and claustrophobic framing create an atmosphere of confusion rather than transcendence. By placing miracle discourse, media circulation, bodily crisis, and public performance within the same narrative world, the film weakens any clear separation between sacred space and the domains of desire, shame, violence, and spectacle. By positioning religious spectacle alongside bodily excess and emotional panic, the film exposes how patriarchal moral systems themselves generate instability and fear within contemporary social life. The film also critiques blind faith and moral irrationality through dialogue associated with the miraculous child narrative. During moments of collective hysteria surrounding the child’s supposed divinity, ordinary suffering and emotional vulnerability become secondary to spectacle and public obsession. The repeated public reactions, devotional behaviour, and media circulation surrounding the “miracle” reveal how spirituality becomes commodified through mass emotional performance rather than ethical engagement. In this context, religion functions not as moral guidance but as another unstable institution participating in the circulation of fear, social anxiety, and symbolic contamination throughout the urban environment.

### 5.2 Queer bodies and social abjection

Shilpa’s return makes visible the mechanisms through which heteronormative society produces queer abjection through public scrutiny, familial discomfort, bureaucratic exclusion, and the policing of “normality.” Drawing on Kristeva’s concept of abjection and Douglas’s account of impurity as “matter out of place,” this section examines how the film frames transgender embodiment not as inherently contaminating, but as socially cast as disruptive when it unsettles the classificatory boundaries of gender, parenthood, and domestic respectability.

#### 5.2.1 Public disgust and the spectacle of difference

The film immediately frames Shilpa’s return as a socially disruptive spectacle through public reactions marked by shock, ridicule, and disbelief. During Shilpa’s entry into the neighbourhood, surrounding characters respond with visible disgust and confusion:

*I can't believe my eyes!*

*“What the hell is this?”* (Super Deluxe, 2019, 00:23:16–00:23:19)

Moments later, another character reacts:

*“Has he gone mad or what?”* (Super Deluxe, 2019, 00:23:34–00:23:36)

These responses immediately position Shilpa’s transformed identity as socially unintelligible within the heteronormative environment. The language used by the surrounding characters reflects what Douglas (1966) identifies as cultural anxiety toward bodies that blur established social categories. The issue within the scene is not merely gender transition itself, but the collapse of familiar social classifications through which patriarchal society organizes identity and normalcy. Visually, the scene intensifies this discomfort through observational framing, crowded public space, and prolonged reactions from surrounding characters. The camera repeatedly captures staring faces, hesitant bodily gestures, and emotional unease, transforming the street into a space of surveillance and social judgment. However, rather than depicting Shilpa as monstrous or threatening, the film redirects attention toward the discomfort of the crowd itself. Public disgust therefore becomes a reflection of collective social insecurity rather than evidence of queer deviance.

#### 5.2.2 Familial instability and gendered humiliation

Shilpa’s return destabilizes not only public space but also the emotional structure of the family. Her wife’s suffering becomes framed through the language of social shame, emotional abandonment, and heteronormative collapse. One character painfully remarks:

*“As a mother, I shouldn't say this. Instead of waiting for him, you could've eloped with another man.”* (Super Deluxe, 2019, 00:49:32–00:49:39)

The dialogue reveals how transgender identity becomes interpreted through the lens of familial failure and social humiliation. The scene frames marriage and domesticity through expectations of stable gender performance, so Shilpa’s transition becomes legible to the family as a disruption of the roles through which heterosexual kinship has been organized. The emotional pain expressed within the scene emerges not simply from personal betrayal but from the collapse of normative expectations surrounding masculinity, husbandhood, and social respectability. Raasukutti’s confusion regarding familial identity further reveals the instability of gendered social categories. He hesitantly asks that, *“Now that you are a woman, should I call you Dad or Mom?”* (Super Deluxe, 2019, 01:18:40–01:18:47). The dialogue becomes significant because it exposes the inability of heteronormative language to accommodate identities existing outside rigid binary classifications. Rather than mocking Shilpa, the child’s question reflects an attempt to emotionally reorganize familial relationships beyond conventional gender structures. The scene therefore reveals how patriarchal society depends upon stable linguistic categories in order to maintain normative ideas of family, parenthood, and identity. At the same time, the film complicates this instability through the perspective of Raasukutti, whose acceptance of Shilpa contrasts sharply with the discomfort of surrounding adults. When Shilpa asks,

*“Why? Do not you like it?”* (01:16:14).

Raasukutti responds,

*“No, it’s nice. I like it. I really do.”* (Super Deluxe, 2019, 01:16:16–01:16:18).

This exchange becomes deeply significant because it interrupts the social logic of disgust operating throughout the narrative. Unlike adults shaped by patriarchal morality and rigid gender structures, the child responds without moral panic or symbolic anxiety. Acceptance therefore emerges not from social conditioning but from emotional intimacy and human connection.

#### 5.2.3 Queer identity and the politics of “normality”

One of the film’s most explicit critiques of heteronormative social order appears through repeated references to the idea of “normality.” During the police station sequence, an officer remarks: *“Next time you take your son out, take a normal person along.”* (Super Deluxe, 2019, 01:33:10–01:33:14). This line becomes central to the film’s critique of normative social structures because it openly constructs queer existence as incompatible with social normalcy. The statement reflects what queer ecological scholars identify as the ideological connection between heterosexuality and “naturalness,” where socially accepted identities are framed as biologically and morally legitimate while queer bodies become associated with disorder or abnormality (Mortimer-Sandilands and Erickson, 2010). A similar moment occurs during the school confrontation scene, where the guard states, “*This thing cannot be your father.”* (01:53:04–01:53:07). The dehumanizing use of “this thing” reflects the extreme form of social abjection imposed upon transgender bodies. Shilpa becomes linguistically displaced from personhood itself, reinforcing Kristeva’s argument that abjection operates through the symbolic expulsion of bodies perceived as threatening to social order (Kristeva, 1982). The school space therefore functions as an institutional environment where normative identity is regulated through humiliation and exclusion. However, the film simultaneously destabilizes the meaning of “normality” itself. During a conversation with his friends, Raasukutti responds: “*Why cannot dads look like moms?”* (Super Deluxe, 2019, 01:56:30–01:56:32). This simple yet powerful question exposes the arbitrary nature of gender norms by revealing how social discomfort emerges not from the body itself but from culturally conditioned expectations surrounding masculinity and family structure.

#### 5.2.4 Abjection, resistance, and queer self-articulation

The film reaches its strongest articulation of queer resistance during the conversation between Shilpa and the granny. When questioned about whether her child can live a “normal life,” Shilpa responds:

*“What wrong have I done? Don't we cut our nails? Don't we cut our hair to suit our taste? Similarly, I altered my body a bit. Where's the crime in that?”* (Super Deluxe, 2019, 01:55:05–01:55:11).

This dialogue is significant because it reframes bodily transformation because it reframes bodily transformation as an ordinary form of self-fashioning rather than moral deviance. The comparison between grooming practices and gender transition destabilizes rigid distinctions between “natural” and “unnatural” bodies, thereby challenging heteronormative assumptions surrounding bodily legitimacy. Shilpa’s later advice to Raasukutti further exposes the violence of social conformity:

*“Live exactly how the world wants you to. Don’t think originally. Don’t be unique. Blend with the crowd. Uniqueness is feared by the world. Fear leads to hatred.”* (Super Deluxe, 2019, 01:59:17–01:59:33).

The dialogue directly articulates the social mechanisms through which nonconforming identities become marginalized, feared, and excluded. Difference itself becomes threatening because it destabilizes collective systems of social order and patriarchal normalcy. Yet the film ultimately rejects exclusion through the emotional reconciliation between Shilpa and Raasukutti. In one of the film’s most powerful moments, Raasukutti tells Shilpa that, *“Be a man or be a woman, just be with us.”* (Super Deluxe, 2019, 02:37:08–02:37:11). This line functions as the emotional and ideological resolution of the narrative. Rather than demanding conformity to normative gender categories, the scene prioritizes emotional presence, care, and relational belonging over patriarchal definitions of identity. The film therefore transforms queer abjection into a site of resistance by exposing the instability of the very social structures that produce exclusion.

### 5.3 Relocating impurity: society as contamination

One of the film’s most significant gestures lies in the way it gradually redistributes exclusion away from Shilpa alone and across the institutions, domestic structures, and moral performances that seek to regulate her. The neighbourhood’s shocked response to her return, the school watchman’s refusal to recognize her parental legitimacy, the police officer’s “normal person” remark, and the grandmother conversation about bodily alteration all show how queer embodiment becomes the immediate target of scrutiny. Yet, when these scenes are read alongside the film’s other narrative strands especially those involving secrecy, male panic, religious spectacle, pornography, and emotional violence the source of instability no longer appears to reside in Shilpa alone. Instead, the film repeatedly situates shame, coercion, humiliation, and moral contradiction within the very structures that claim to defend normalcy. The recurring use of cluttered interiors, decaying urban corners, public thresholds, and emotionally tense domestic spaces strengthens this reversal by embedding social regulation within a visibly unstable urban world. In this sense, *Super Deluxe* does not simply attach stigma to queer embodiment; it increasingly redirects symbolic pollution toward the heteronormative institutions, patriarchal performances, and moral systems that produce exclusion in the first place. The film repeatedly presents recurring urban settings, including cluttered streets, waste-adjacent spaces, dim corridors, and isolated urban locations. These environments reinforce the film’s recurring themes of exposure, vulnerability, and compromised belonging.

### 5.4 Hyperlink narrative and ecological interconnectedness

One of the most distinctive features of *Super Deluxe* is its hyperlink narrative structure, through which multiple seemingly disconnected storylines gradually intersect within the same fragmented urban environment. Rather than functioning as isolated narratives, the film’s interconnected character arcs collectively construct Chennai as an unstable social ecology where violence, shame, desire, secrecy, morality, and emotional instability continuously circulate across different spaces, institutions, and bodies. Hyperlink cinema frequently emphasizes fragmentation, simultaneity, and interdependence in order to represent the complexity of contemporary urban life (Bordwell, 2008). In the film, however, this fragmentation does not produce narrative separation; instead, it exposes the ecological interconnectedness of social experiences operating within the city. Queer identity, pornography, religious spectacle, domestic violence, masculinity, media culture, police authority, emotional repression, and bodily anxiety all circulate through overlapping narrative pathways that repeatedly collapse distinctions between public and private life. The film therefore presents urban social life as relationally interconnected, with shame, secrecy, desire, and moral anxiety moving across narrative strands rather than remaining confined to a single character or household. Violence in one storyline echoes through another; shame becomes socially transferable; secrecy circulates across domestic, institutional, and public spaces; and desire repeatedly destabilizes structures of normative control. The hyperlink structure also connects sexual secrecy, bodily exposure, and moral anxiety across otherwise separate narrative strands. Scenes involving pornography, motel encounters, secrecy, and emotional panic do not remain confined to a single storyline; instead, they echo the film’s broader atmosphere of shame, surveillance, and unstable respectability. Read alongside Shilpa’s experiences of public scrutiny and institutional exclusion, these moments suggest that desire in *Super Deluxe* is repeatedly mediated through concealment, discomfort, and social regulation rather than through privacy or intimacy. In this way, the film presents stigma as something that circulates across interconnected spaces of desire, secrecy, and moral judgement rather than as a stigma attached to one body alone.

### 5.5 Patriarchal masculinity, repression, and urban instability

Ecofeminist and queer ecological scholarship frequently identify patriarchal systems as structures that depend upon domination, control, hierarchy, and exclusion, often producing forms of ecological and social violence simultaneously (Gaard, 1997; Alaimo, 2010). One of the film’s most important interventions lies in its ecological representation of patriarchal masculinity as a destructive social force circulating through the urban environment. Emotional repression, violence, domination, secrecy, and moral policing repeatedly contaminate domestic spaces, institutions, and interpersonal relationships, creating an unstable social ecology shaped by fear and fragmentation. This ecological collapse becomes visible through the film’s recurring aesthetics of urban decay, bodily discomfort, fragmented interiors, polluted environments, and emotional suffocation. Masculinity repeatedly contributes to these conditions by sustaining systems of repression and instability rather than ethical or emotional coherence. Across the film’s narrative strands, scenes of male aggression, concealment, and emotional repression recur alongside cramped interiors, conflict-ridden domestic spaces, and unstable urban settings. Read together, these patterns link patriarchal control to a broader atmosphere of shame, fear, and relational breakdown rather than to moral authority or stability. Importantly, the film contrasts this violence with moments of emotional vulnerability, acceptance, and relational intimacy, particularly through the interactions involving Shilpa and Raasukutti. These moments suggest alternative forms of relational existence that reject domination and rigid gender hierarchy. The film therefore critiques patriarchal masculinity not simply as an individual behavioural problem but as a broader ecological system that produces emotional destruction across urban life. By linking toxic masculinity with violence, repression, and ecological fragmentation, the film ultimately demonstrates that patriarchal social structures themselves function as contaminating forces within contemporary urban society. Ecological collapse within the film is therefore not limited to polluted environments or urban decay alone; it also emerges through the emotional and psychological damage produced by systems of masculine domination and heteronormative control.

## 6 Discussion

### 6.1 Queer ecology and impurity in Indian cinema

This study contributes to the growing field of queer ecology by extending its analytical focus beyond the relationship between sexuality and nature to include the ecological and spatial significance of urban environments, institutional structures, and everyday social spaces. While queer ecology has primarily examined how ideas of nature, purity, and normality sustain heteronormative assumptions (Mortimer-Sandilands and Erickson, 2010), the present study demonstrates that these regulatory logics also operate through the material and symbolic organization of the postcolonial city. Rather than treating urban space as a neutral backdrop, *Super Deluxe* presents the city as an active environment through which exclusion, surveillance, and moral regulation are produced and experienced. Existing scholarship on Indian queer cinema has predominantly emphasized questions of representation, identity, visibility, and social acceptance. By contrast, this study argues that queer embodiment is also shaped through spatial arrangements, environmental conditions, and institutional practices that collectively influence how gender and sexuality are publicly negotiated. In this respect, the ecological and spatial dimensions of queerness extends beyond the natural environment to encompass the interconnected relationships among bodies, urban infrastructures, domestic spaces, and social institutions. Accordingly, the contribution of this study is not simply that it offers another interpretation of *Super Deluxe*, but that it demonstrates how queer ecology can be productively applied to South Asian urban cinema. The findings suggest that ecological thinking provides a broader analytical vocabulary for understanding how sexuality is regulated through space, affect, and everyday urban life, thereby expanding the scope of queer ecological scholarship beyond its traditional emphasis on wilderness, rural landscapes, and environmental conservation. By situating queer embodiment within the material conditions of the contemporary city, the study highlights the importance of urban ecologies as critical sites for examining the intersection of sexuality, social exclusion, and environmental experience in postcolonial contexts.

### 6.2 Ecologies of impurity as a conceptual framework

A central contribution of this study is the concept of Ecologies of Impurity, proposed as a framework for understanding how abjection is produced through the interaction of bodies, urban spaces, institutions, and moral systems rather than being located within marginalized identities alone. While developed through the analysis of *Super Deluxe*, the framework is intended as a broader analytical approach for examining how environmental, spatial, and social processes collectively shape experiences of exclusion. The framework builds upon, but differs from, existing theories. Whereas queer ecology primarily examines how ideas of nature and normality regulate sexuality, Ecologies of Impurity shifts attention to the urban environments and institutional spaces through which these norms are enacted. Likewise, Kristeva’s theory of abjection explains how societies construct boundaries by excluding what is considered impure, whereas the present framework extends this insight by examining how exclusion is materially organized through urban infrastructures, domestic spaces, and public institutions. Rather than replacing these theories, Ecologies of ecological process synthesizes their insights to explain how exclusion is produced through the interaction of spatial, ecological, institutional, and social processes, thereby providing an integrated framework for analysing exclusion in urban cultural contexts. The broader value of this framework lies in its applicability beyond *Super Deluxe*. It offers a conceptual approach for analysing cinema, literature, and other cultural texts that explore the intersections of sexuality, urban environments, institutional regulation, and moral discourse. By locating marginalization within ecological and social systems rather than within queer bodies themselves, the framework contributes to ongoing discussions in queer ecology, urban ecocriticism, environmental humanities, and Global South film studies. As an analytical framework, Ecologies of Impurity enables researchers to examine how environmental conditions, institutional practices, and spatial organization collectively shape processes of social exclusion across diverse cultural contexts.

### 6.3 Implications for urban ecocriticism and global south queer studies

The present study contributes to urban ecocriticism by demonstrating that cities are not merely physical environments but also spaces where ecological conditions, institutional practices, and social norms intersect to shape experiences of inclusion and exclusion. In *Super Deluxe*, urban environments are shown to participate in the production of social meaning through domestic spaces, public institutions, and everyday infrastructures, suggesting that environmental and social marginalization are closely interconnected. This perspective expands urban ecocritical scholarship by highlighting how sexuality and moral regulation are embedded within the material organization of the postcolonial city. The study also contributes to Global South queer studies by situating queer experience within the specific cultural, religious, and institutional contexts of contemporary India. Rather than treating queer exclusion as a universal phenomenon, the analysis demonstrates how local social structures, familial expectations, public respectability, and institutional authority shape the lived experience of gender and sexual diversity. In doing so, the study emphasizes the importance of examining queer identities through regionally grounded ecological and spatial frameworks. Beyond *Super Deluxe*, the framework proposed in this study encourages future research to examine how environmental conditions, urban infrastructures, and institutional spaces influence representations of gender, sexuality, and social belonging across other South Asian films and cultural texts. Such an approach provides opportunities for greater dialogue between queer ecology, urban ecocriticism, environmental humanities, and film studies, particularly in contexts where ecological degradation and social marginalization remain deeply interconnected.

## 7 Limitations

While this study provides a detailed queer ecological reading of *Super Deluxe*, several limitations should be acknowledged. First, the analysis focuses on a single Tamil film, limiting the broader generalizability of the findings across Indian cinema. Although *Super Deluxe* offers a rich narrative for examining the intersections of sexuality, urban space, and moral regulation, other regional cinemas may represent these themes through different cultural, aesthetic, and ideological perspectives. Accordingly, the study should be understood as an in-depth interpretive analysis rather than a comprehensive account of queer ecological representation in Indian cinema. Second, the study adopts a qualitative approach based on narrative, visual, and cinematic analysis. While close textual analysis enables a detailed examination of the film’s spatial, visual, and thematic dimensions, it does not incorporate audience reception, interviews, ethnographic research, or filmmaker perspectives. Consequently, the study cannot assess how different audiences interpret or negotiate the film’s representations of queerness and urban space. Third, although the analysis examines sexuality, urbanity, and institutional regulation, it does not explore the intersections of caste, religion, class, labour precarity, or disability in depth. Future intersectional research may provide a more comprehensive understanding of how multiple systems of marginalization interact within postcolonial urban environments. Finally, as with all qualitative interpretive research, the analysis is shaped by the selected theoretical frameworks of queer ecology, urban ecocriticism, abjection theory, and Global South queer studies. To enhance analytical credibility, the study employed repeated film viewings, a systematic scene-based analytical process, reflexive validation, constant scene comparison, an audit trail, and theoretical triangulation. Nevertheless, the interpretation remains informed by the researcher’s analytical perspective, and alternative theoretical frameworks may generate different readings of the film’s visual, narrative, and symbolic dimensions.

## 8 Future scope

This study opens several avenues for future research within queer ecology, South Asian cinema studies, environmental humanities, urban cultural studies, and Global South queer scholarship. Future studies may extend this analysis by examining a broader corpus of Indian regional films to explore how different linguistic, cultural, and cinematic traditions negotiate the relationships among sexuality, urban environments, institutional regulation, and moral discourse. Further research may also incorporate audience reception, ethnographic methods, interviews with filmmakers, or LGBTQ+ communities to investigate how cinematic representations of queer identities are interpreted, negotiated, and experienced across diverse social contexts. Such approaches would complement textual analysis by connecting cinematic representation with lived experience. In addition, future scholarship may adopt a more intersectional perspective by examining how caste, religion, class, disability, labour precarity, and digital culture interact with sexuality and urban ecology within postcolonial contexts. Comparative studies involving South Asian and international cinema may further illuminate how different cultural settings negotiate questions of space, morality, exclusion, and belonging. Finally, the concept of Ecologies of Impurity may be further refined and applied to literature, television, digital media, visual culture, and other cultural texts. Extending this framework across diverse media and geographical contexts would contribute to a broader interdisciplinary understanding of how environmental conditions, institutional structures, and moral systems collectively shape processes of social exclusion.

## 9 Conclusion

This study examined *Super Deluxe* through the intersecting frameworks of queer ecology, urban ecocriticism, abjection theory, and Global South queer studies to investigate how contemporary Tamil cinema constructs the relationship between sexuality, urban space, and moral regulation. Moving beyond representational approaches to queer identity, the analysis demonstrated that the film locates queer experience within interconnected urban environments, institutional structures, and everyday social spaces, showing how exclusion is produced through spatial, ecological, and social processes rather than through identity alone. The principal theoretical contribution of this study is the development of the concept of Ecologies of Impurity. Building upon queer ecology, urban ecocriticism, and Kristeva’s theory of abjection, this framework explains how processes of exclusion are produced through the interaction of bodies, urban infrastructures, institutions, and moral systems. By conceptualizing marginalization as an ecological and spatial process, the framework provides a broader analytical approach for examining how environmental conditions and social structures collectively shape experiences of exclusion.

The study also contributes to queer ecology by extending its analytical scope beyond the relationship between sexuality and nature to include the ecological significance of postcolonial urban environments. At the same time, it contributes to Global South film scholarship by demonstrating how regionally specific cultural, religious, and institutional contexts shape the ecological dimensions of queer experience within contemporary Indian cinema. These findings encourage greater dialogue between queer ecology, urban ecocriticism, environmental humanities, and film studies. Although the framework has been developed through the analysis of *Super Deluxe*, its broader value lies in its potential application to other films, literary texts, and cultural narratives that explore the intersections of sexuality, urban environments, institutional regulation, and moral discourse. Future research may further refine and extend the concept of Ecologies of Impurity across different cultural, geographical, and media contexts, contributing to interdisciplinary scholarship on ecology, sexuality, space, and social justice.

## Data Availability

The original contributions presented in the study are included in the article/supplementary material, further inquiries can be directed to the corresponding author.
